# Development and Physico-Chemical Characterization of a Shea Butter-Containing Lipid Nutrition Supplement for Sub-Saharan Africa

**DOI:** 10.3390/foods6110097

**Published:** 2017-11-08

**Authors:** Elizabeth M. Sloffer, Shashank Gaur, Nicki J. Engeseth, Juan E. Andrade

**Affiliations:** 1 Department of Food Science and Human Nutrition, University of Illinois, Urbana-Champaign, Urbana, IL 61801, USA; sloffer2@illinois.edu (E.M.S.); gaur2@illinois.edu (S.G.); engeseth@illinois.edu (N.J.E.); 2 Division of Nutritional Sciences, University of Illinois, Urbana-Champaign, Urbana, IL 61801, USA

**Keywords:** lipid-based nutrition supplement, shelf-life, malnutrition, food security, food aid, Shea butter, flaxseed oil, ready to use supplementary food

## Abstract

Lipid-based nutrient supplements (LNS) are used to prevent and treat moderate and severe acute malnutrition, a leading cause of mortality in children-under-five. The physical and chemical changes of two new LNS products were evaluated before and after accelerated shelf life testing (ASLT) according to protocols suggested by the U.S. Agency for International Development (USAID) and Doctors without Borders and compared against USAID’s A-20 paste as a control. LNS formulas containing Shea butter from the Shea nut tree (*Vitellaria paradoxa*), a common fat source in parts of Sub-Saharan Africa, with and without flax-seed oil, as a source of omega-3 fatty acids, were developed. LNS formulas were batched (0.8 kg) in a wet grinder, sealed under nitrogen in three-layer mini-pouches (20 g), and underwent ASLT at 40 ± 2 °C for six months with sampling every eight weeks. At each time point, water activity, moisture, peroxide value, oil separation, vitamin C content, and hardness were evaluated. Results showed comparable stability among all formulas with an increase in Aw (*p* < 0.05) but no change in vitamin C, oil separation, or peroxide value. Addition of Shea butter improved the LNS’s hardness, which remained stable over time. Modifying fat profile in LNS can improve its texture and essential fatty acid content without affecting its storage stability.

## 1. Introduction

In 2015, 45% of the nearly 6 million deaths among children under 5 were due to nutrition-related illnesses [1]. Although the prevalence of acute and chronic malnutrition in children under five years of age continues to decline, large numbers of children remain malnourished especially in South and Central Asia and Sub-Saharan Africa. Acute malnutrition or wasting manifests in low weight for height. Moderate acute malnutrition (MAM) is defined when children’s weight for height is between −2z and −3z scores below the median of the World Health Organization (WHO) growth standards. Severe acute malnutrition (SAM) is defined when children’s weight for height falls below −3z scores below the median of the WHO growth standards, along with other signs such as emaciation and edema [2]. Malnutrition and its associated diseases continue to be a serious problem, resulting in significant research efforts on effective preventive and control measures that support nutrition interventions.

Historically, treatment of SAM includes the use of F-75 and F-100 fortified milk formulas. F-75 is a relatively low calorie (75 kcal/100 mL), low protein (0.9 g/100 mL) formula provided in health centers to the most malnourished children presenting no appetite and other complicating conditions, such as edema or fever [3]. Children are then transitioned to the F-100 formula which contains more calories (100 kcal/100 mL) and protein (2.9 g/100 mL). The major drawback of F-75 and F-100 is that they are powdered fortified milk-based formulas which must be combined with clean water and have a shelf life of only a few hours after preparation [4,5,6]. This means that they can only be safely administered in a clinical setting. However, children who do not present with serious symptoms of appetite loss and edema may be treated with ready-to-use therapeutic foods (RUTF) in the community rather than clinical setting. RUTF are classified as foods used to address malnutrition that do not require preparation at home beyond opening and consuming the food. Frequently, these foods take the form of cookies and bars. Common lipid-based RUTFs used in the management of SAM are Plumpy’Nut and Nutributter [7].

LNSs typically contain the same protein, carbohydrate, and micronutrient profiles as F-75 and F-100 fortified milk formulas, but the lipid content is increased to become the primary matrix and source of calories of the product. LNSs are ready to eat, an advantage because they do not require any preparation or a clean water source. Due to their low water activity, they have a long shelf life of 1–2 years without refrigeration and risk of contamination when properly packaged and stored. This enables the treatment of SAM in the community setting, away from clinics [5,6]. The first, peanut-based, LNS products were developed and evaluated in the late 1990s as RUTFs to treat marasmus [8,9]. LNS have since gained recognition as an effective therapy to treat SAM, in the prevention of child stunting, and the support of normal growth and development as shown in trials in Malawi and Ghana [5,10,11]. The advent of LNS advanced the treatment of malnutrition and it continues to be one of the most effective treatments for acute malnutrition. Beyond acute malnutrition, the use of small quantity LNS has been evaluated to complement existing food strategies aimed at preventing stunting in low-income settings as reviewed by Matsungo et al. [12]. Additionally, the controversy over the patenting of these products remains an issue because of licensure requirements [13]. As such, development of alternative formulas, which can be used in small quantities to complement meals or as a complete meal replacement, avoiding patented ingredients, like peanuts, is desirable.

To date, most literature on lipid nutrition supplements (LNS), commonly published in pediatric health and nutrition journals, is aimed at measuring the efficacy of treatment schemes. While critical, this fails to develop our understanding of LNS’ characteristics as food products in their own right.

The United States Agency for International Development (USAID) developed its A-20 LNS formula following the specifications set forth by the Subcommittee on Technical Specifications for a High-Energy Emergency Relief Ration [14]. However, the product hardens and may separate during storage [15]. In addition, the product is also not very palatable, and previous research on LNS shows that an unappetizing product may compromise patient adherence to treatment [16,17]. An additional, important drawback is that A-20 and similar products do not supply a balanced amount of polyunsaturated (i.e., ω-3 and ω-6) fatty acids, which are important for healthy brain development and controlling inflammation [18,19,20]. A reformulated LNS made with local ingredients might be more acceptable and, therefore, have better patient adherence.

In this study, two new LNS products were created using canola oil, flaxseed oil as a source of ω-3 fatty acids, and Shea butter (*Vitellaria paradoxa*), a commonly consumed dietary fat in Western Sub-Saharan Africa. These LNS products were characterized and compared to USAID’s A-20 LNS as a control in terms of physical and chemical parameters before and after ASLT according to the method employed by USAID [15]. The hypothesis tested was that by completely changing the lipid system in USAID’s A-20 LNS, a LNS with similar stability to A-20 would be created with enhanced texture and ω-3/ω-6 fatty acid balance.

## 2. Materials and Methods

### 2.1. Ingredients, Chemicals, and Materials

Food grade materials were obtained from local and online retailers: confectionary sugar, soybean oil, canola oil, salt, (Great Value, Bentonville, AR, USA), non-fat dry milk (Farm, Cridersville, OH, USA), cocoa powder (Hershey’s, Hershey, PA, USA), whey protein concentrate (Hilmar Ingredients, Hilmar, CA, USA), dried cream (Ciao Imports, Miami Beach, FL, USA), Shea butter (Essential Depot, Sebring, FL, USA), vitamin mineral premix (Watson, Inc., West Haven, CT, USA), soy lecithin, pea protein (Now Foods, Bloomingdale, IL, USA), Ascorbyl Palmitate (Spectrum Chemicals Corp, New Brunswick, NJ, USA), butylated hydroxyl anisole (BHA), mixed tocopherol (Sigma-Aldrich, St. Louis, MO, USA), flax seed oil (Spectrum Organic Products, Melville, NY, USA), and maltodextrin (Tate and Lyle, London, UK). Three-layer mini-pouches (PAKVF4) were purchased from IMPAK (Los Angeles, CA, USA).

Whenever possible, HPLC-grade reagents and solvents were purchased. These included hexane, acetic acid, potassium iodide, sodium thiosulphate, and soluble starch solution from Fisher Scientific (New Brunswick, NJ, USA) as well as chloroform, metaphosphoric acid, ethylenediaminetetraacetic acid, sodium phosphate monobasic and L-ascorbic acid from Sigma-Aldrich (St. Louis, MO, USA).

### 2.2. Recipe Formulation

Formulations for LNS were designed using ESHA Food Processor Diet Analysis and Fitness Software v. 10.10.0 (ESHA Research, Salem, OR, USA) and the United States Department of Agriculture (USDA) nutrient database [21]. Calculations were set to yield approximately 510–520 kcal, 11–12% protein from dairy and pea; 40–45% carbohydrate from sucrose, maltodextrin and other ingredients; 32–36% fat from canola oil, soybean oil, Shea butter, and flax seed oil; and 3% complete vitamin and mineral mix as shown in Table 1. Cocoa powder (10%) was added to new formulas for flavoring.

New formulas were designed to match or exceed nutritional specifications of USAID’s A-20 formula [15]. Importantly, A-20 does not meet World Food Program (WFP) and MSF/UNICEF specifications because of the use of butylated hydroxyanisole (BHA) as a preservative [22,23]. The 2009 USDA Commercial Item Description for Emergency Food allows the use of natural and artificial preservatives including BHA [24], and available documents specifying the labeling of A-20 also include BHA [15,25]. However, the 2012 USDA Commercial Item Description for RUTF prohibits the use of artificial antioxidants and flavorings [24]. BHA was kept as an ingredient in these formulations to compare it with the most complete information available on the formulation of A-20 available in the original report.

### 2.3. Production and Storage of LNS

**Figure 1:**
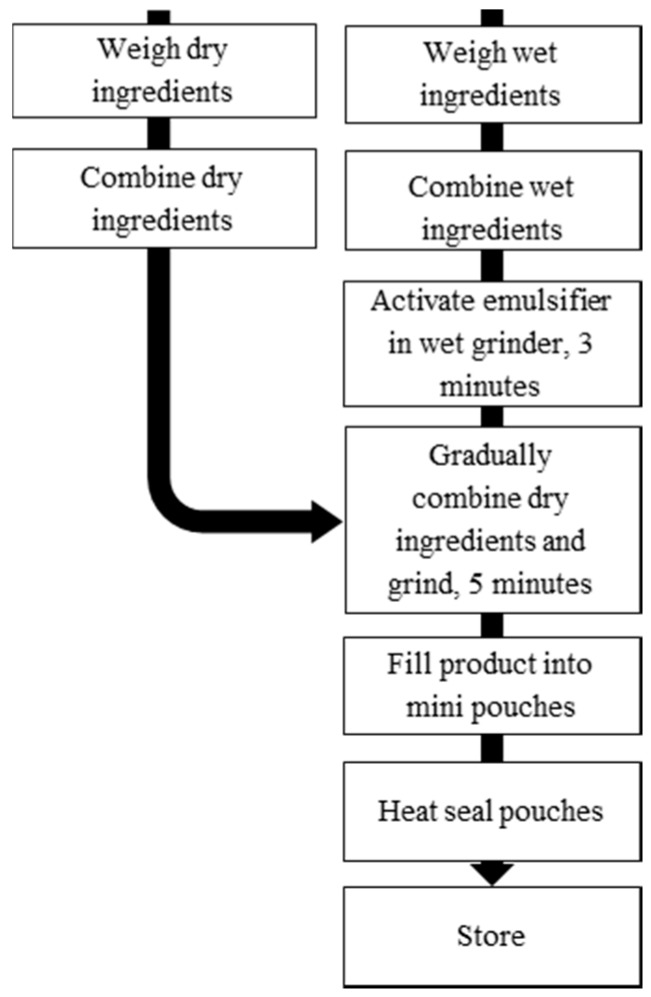
Lipid-based nutrient supplements (LNS) processing flow diagram.

See Figure 1 for complete process flow diagram. Three batches (0.8 kg) of each LNS formula (Table 1) were made to complete all analyses in triplicate. Solid fat was melted at 70 °C using a double boiler on an electric hot plate, which was then combined with the oils, mixed tocopherols, and lecithin. Flax seed oil was added immediately prior to adding into the wet grinder in order to preserve the oil. Lipids were mixed in the wet grinder for 3 min to activate the emulsifier. Gradually, dry ingredients were added to the wet grinder until completely combined, scraping the sides with a spatula as necessary. The LNS was mixed an additional 5 min to ensure homogeneity.

LNS was packaged in three-layer mini-pouches and heat-sealed under ultra-pure nitrogen (99.99%), using a Minipack America Sealer (MVS 31–XP; Minipack, Orange, CA, USA) with the following settings: vacuum 50%, gas ratio 20% and time 2.5 s. The sealed pouches were held at 40 ± 2 °C for six months, according to methods recommended by USAID and MSF for accelerated shelf life testing of LNS [15,23]. Samples were taken at 0, 2, 4, and 6 months for evaluation. Analyses were conducted in triplicate.

### 2.4. Water Activity

Water activity was determined using an Aqualab water activity meter [26] (Aqualab, Pullman, WA, USA). Sample aliquots (1 g) were weighed into 15 mL disposable sample cups (diameter 1.53 cm and height 0.447 cm).

### 2.5. Peroxide Value

The peroxide value was attained according to Crow and White [27], using 2 g of each product. A small number of samples (*n* = 5) were tested using the larger volume method to confirm results [28].

### 2.6. Moisture Content

Moisture content was determined using a CEM microwave moisture analyzer (Smart 6, CEM Corp., Matthews, NC, USA) for 7 min at 25% power and after spreading the sample (2.5 g) evenly over the fiberglass pads.

### 2.7. Lipid Separation

Lipid separation was measured in triplicate using a modified method from Hinds et al. [29]. Pouches were thoroughly and uniformly massaged for 2 min prior to carefully transferring 30 g to a 50 mL centrifuge tube and spinning at low-speed (300× *g*) for 3 min at room temperature (22 °C) (ST 16R Sorvall Centrifuge, TX400 swinging bucket rotor, Thermo Sci., Waltham, MA, USA). A digital Vernier caliper was used to determine the height of lipid layer separated at the top of the sample.

### 2.8. Texture Analysis

Sample hardness was measured using a texture analyzer (TA-XT2; Texture Technologies Corp., Scarsdale, NY, USA) following a method adapted from Ahmed and Ali [30]. Pouches extracted from incubator were thoroughly and uniformly massaged for 2 min, and a 30 g sample was placed in Petri dishes (15 cm diameter and 1.5 cm deep). Samples were allowed to set undisturbed for 1 h, and the temperature was monitored using a handheld thermometer. The sample dish was secured to the base during the analysis. A 50 mm diameter stainless steel, flat-surface probe was fitted into the crosshead of the texture analyzer. The probe was moved at a crosshead speed of 0.5 mm/s with 4 mm penetration. Hardness (kg) was measured as the peak force of the first compression cycle.

### 2.9. Vitamin C

A modified method of Tarrago-Trani et al. [31] was used for extraction and measurement of vitamin C. A 0.2 g sample was extracted with buffer containing 1 mM EDTA/4% meta-phosphoric acid. Separation and quantification of vitamin C were carried out by reverse phase separation and UV detection (PDA at 265 nm) using a Waters HPLC (Milford, MA, USA) with an isocratic pump system. An ISIS C-18 column (150 × 4.6 mm, 3 μm particle size; Supelco-Sigma, St. Louis, MO, USA) was equilibrated with mobile phase consisting of 25 mM sodium phosphate buffer, pH 3.0 and pumped at 0.4 mL/min. Vitamin C was quantified using HPLC grade external standards. Results were expressed as mg of vitamin C per gram of sample.

### 2.10. Data Analysis

All experiments were conducted in triplicates from three different LNS batches, and the results are reported as mean ± SD. Data were analyzed using repeated measures ANOVA in SPSS (IBM, Armonk, NY, USA). Each formula was analyzed over four time points considering the formula effect, time effect, and formula × time effect interaction effect. Statistical significance was established at an alpha of 0.05.

## 3. Results

**Figure 2:**
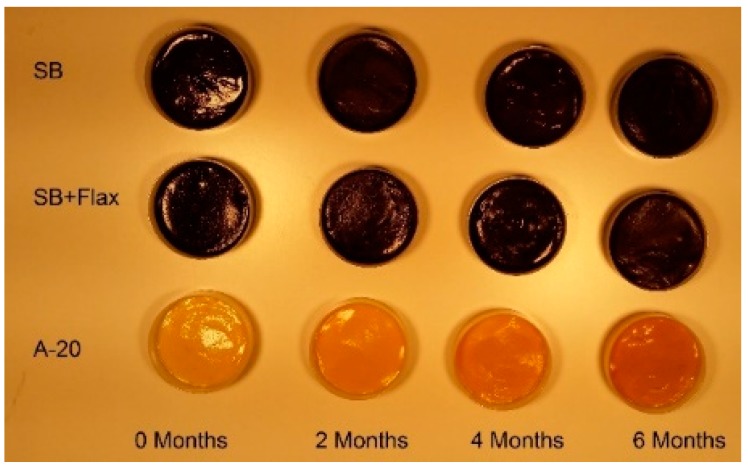
Appearance of LNS products after six months of storage at 40 °C.

**Figure 3:**
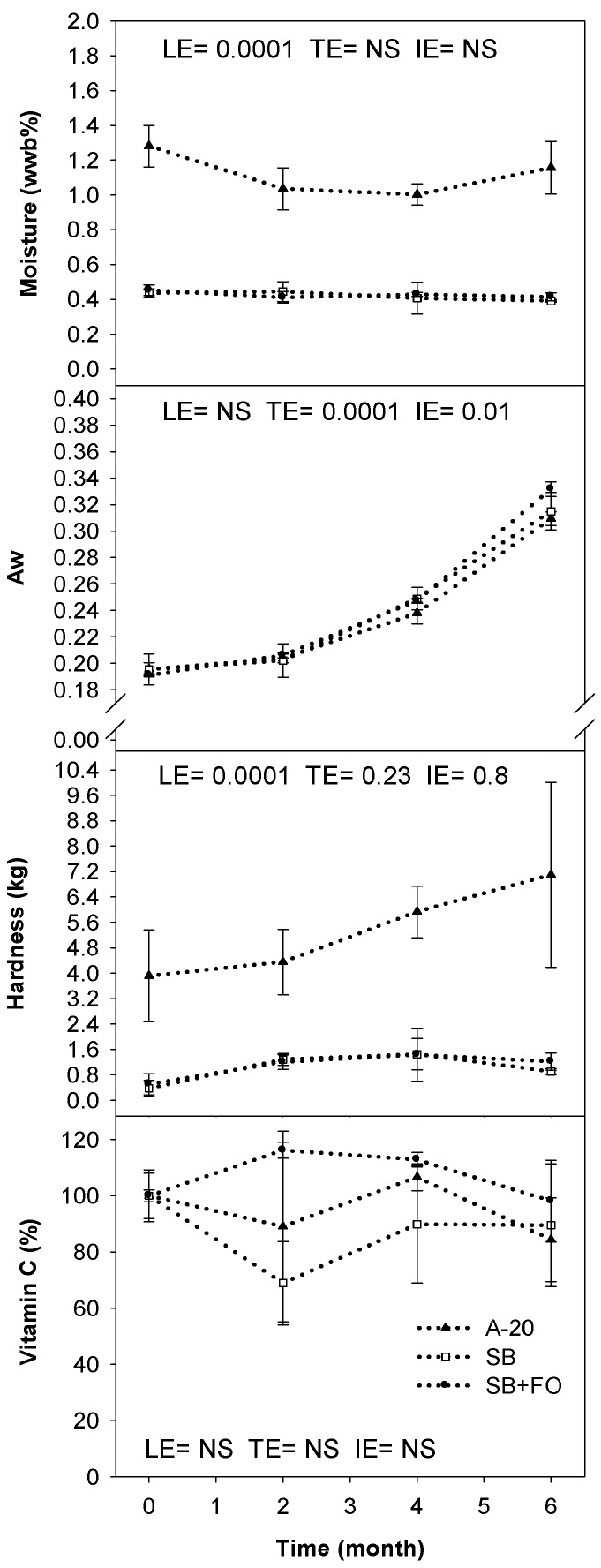
Changes in several chemical and physical parameters in LNS products over six months of storage at 40 °C. Data points represent means ±SD. Factors effects due to LNS (LE), time (TE) and their interaction (IE) were evaluated using repeated measures ANOVA and post hoc mean tests (Tukey HSD). Statistical differences were deemed significant at the level of *p* < 0.05. Non-significant (NS) *p*-values were not included.

Product appearance and changes over time of storage for the A-20, Shea butter (SB), and Shea butter with flax seed oil (SB + F) LNS formulas are presented in Figure 2 and Figure 3. Water activity increased significantly over time in each product (*p* = 0.0001), but there was no significant difference in water activity between the three products. Moisture content (% w.b.) did not change over time, but moisture content was higher (*p* = 0.0001) in A-20 formula than in the two new LNS products. Peroxide values were below the detection limit (<2 mEq/kg) for each sample at any time point. A-20 formula was harder (*p* = 0.0001) than the two new LNS products. Moreover, the hardness in A-20 increased slightly over time, but not significantly. The two new products did not change in hardness over time. No oil separation was observed in any sample at any time. Although vitamin C concentration fluctuated during storage, it did not degrade significantly (interaction effect, *p* = 0.506). There were slight changes in color in A-20 formula (darkening), but not in new LNS products.

## 4. Discussion

### 4.1. Reformulation of LNS

Our team developed two new LNS products using Shea butter and flaxseed oil, which were as stable as the USAID’s A-20 formula, even after the addition of polyunsaturated fatty acids. Criteria for the development of LNS products are available, but these have focused on nutrient and storage requirements [14,32]. LNSs are unusual products in that there are very few products in commercial markets with similar characteristics, the most similar being reduced-fat peanut spread. Reduced-fat peanut spreads are typically made by centrifuging peanut paste to remove fat. Then, a fat replacer is added to restore product volume and texture. In the U.S., the top ingredients used in reduced-at peanut spreads available from national brands are peanut paste and corn syrup solids. This implies that the texture of the food is primarily due to carbohydrates from corn syrup solids and peanut and fat from peanut paste. LNS are similar in that added carbohydrate and fat account for 40–52% and 30–38% of the product formula, respectively. As such, manipulating the carbohydrate and fat content in the LNS formula should have a profound effect on the product’s texture.

Since A-20 and the new LNS formulas have 41–51% carbohydrate, it follows that ingredient selection will be the primary determinant of product texture. A-20 contains maltodextrin and has a harder texture than the new formulas. The use of maltodextrin as a fat replacer in peanut spread has been documented [33]. However, maltodextrin might impart undesirable effects on the texture of reduced fat peanut spreads [34]. Currently, U.S. national brands label corn syrup solids as the primary fat replacer in their reduced fat formulations. This leaves much room for interpretation, as maltodextrin is legally defined as hydrolyzed starch products with <20 dextrose equivalence (DE) and corn syrup solids may be any starch product between 20–95 DE [35]. Dextrose equivalence is based on the degree of hydrolysis of the starch. Pure unhydrolyzed starch has a DE of 0, and pure glucose made from starch is about 95 DE. The use of confectioner’s sugar to replace the maltodextrin in the new formulas is consistent with similar products available on the market.

National brands of reduced-fat peanut spread sold in the U.S. indicate 5–6% saturated fat on the label. Whether a lipid blend behaves mostly as a fat or as an oil is determined by the fatty acid chain length and the number and location of double bonds [36]. The texture or plasticity of fat blends is determined primarily by the fat crystal structure as influenced by its fatty acid content. Plastic fat blends, such as butter or lard, have a desirable, although not fixed, ratio of fatty acids which gives them their distinctive properties [37]. LNS made with pure saturated fat will be very solid while one made with mostly oil will be greasy and may undergo undesirable oil separation and oxidation. As such, increasing the saturation of the lipids in SB and SB + F produced an LNS with a more desirable, softer texture. A-20 gets most of its lipids from soybean oil, in which the predominant saturated fat is C16 palmitic acid. Saturated fat from added lipids is 3.9% of the final A-20 formula. The change to Shea butter resulted in the predominant saturated fatty acid becoming C18 stearic acid. The new formulas contain 4.1% and 4.6% saturated and 16% and 14.2% monounsaturated fat for SB and SB + F, respectively. Flax seed oil contributes additional palmitic acid to formula SB + F as seen in Table 2. Both increasing the total saturated fat and switching to a higher melting point source of saturated fat contribute to a desirable softening of the texture of the two new LNS products.

Shea butter was chosen to replace 5% of the total weight (13% of the lipid content) in new formulas because it is a commonly consumed dietary fat in Western Sub-Saharan Africa [38]. It is also a female-produced, windfall crop. It has been shown that when LNS are not made with local, familiar ingredients, patient adherence to treatment suffers [17]. Shea butter was used to illustrate that any blend of fats may be suitable for use in LNS provided it meets requirements for necessary essential fatty acids and texture.

### 4.2. Characterization and Stability of LNS Formulas

While water activity increased; moisture content did not, indicating that the packaging remained intact and the increase in water activity was simply due to water moving within the product and not related to any compromise of packaging. The higher moisture content of A-20 is attributed to the hygroscopic quality of the maltodextrin used in that formula. The overall water activity for all products remained well below the USDA specification of 0.6 [40].

The antioxidant system (i.e., BHA) was effective in reducing oxidation. The use of artificial antioxidants including BHA is not currently permitted in LNS formulas by USDA, MSF, or WFP [23,40,41,42]. In a previous study, our team demonstrated that lipid oxidation occurred after six months of accelerated storage at 40 ± 2 °C when ascorbyl palmitate, an allowed antioxidant source for the Indian market, was the only antioxidant used and when formulas contained >5% flax seed oil [43]. Flax seed oil will not significantly destabilize an adequately preserved product through oxidation. Additionally, USAID did not report any oxidation in samples containing BHA as well as tocopherol and ascorbyl palmitate [15]. Vitamin C appeared to remain stable in the product at 40 °C. Although unusual, USAID also reported an average loss of 5% of the vitamin C in A-20 paste (21% at 100 °F and 0% loss at 80 °F) in their study—a lower loss than might be expected [15].

There was no oil separation under the ASLT protocol specified by USAID. It is possible that the separation methodology, developed for use in peanut butter, was not harsh enough to evaluate new LNS formulas. Peanut butter is similar in texture to LNS, but differences in emulsifiers and matrix structure could account for the lack of separation in LNS. The softer texture of the new formulas is primarily due to the removal of maltodextrin, as well as the use of both solid fat and oils. It is critical that the product remains soft enough to mix within the pouch in order to guarantee a homogenous distribution of nutrients in each serving in the event separation occurs. Additionally, adequate consistency is important for consumption compliance as thicker products might be too difficult to eat. USAID did not report texture measurements for the A-20 paste [15]. Additionally, the ASLT protocol used might not be optimal for characterizing shelf life in LNS, due to limited capacity for kinetic modelling to predict the end of shelf life. Although color was not objectively assessed in this study, A-20 samples visibly darkened after six months in storage. This change was not as apparent for cocoa-containing LNS products.

## 5. Conclusions

Two new LNS products based on A-20 composition were softer than A-20 but had equal stability. Based on this research, it is recommended to adapt shelf life testing procedures to 60 °C so that modelling for lipid oxidation and vitamin C degradation can be developed for LNS products. The preservatives utilized stabilized unsaturated fatty acids in flax seed oil. Using artificial preservatives, such as BHA, can probably be avoided if shorter shelf lives are tolerable, but long shelf life may be desirable if governments or other entities wish to maintain strategic reserves of LNS products. The risks and benefits of using preservatives in foods designed to deliver high concentrations of unstable nutrients over a long shelf life should be carefully considered. Future research should include sensory evaluation of LNS products with populations in Shea-producing regions of Sub-Saharan Africa.

## Figures and Tables

**Table 1:** Nutrient and ingredient composition of LNS products.

	A-20			SB			SB + F		
Ingredient	Wt (g)	Calories (kcal)	% kcal	Wt (g)	Calories (kcal)	% kcal	Wt (g)	Calories (kcal)	% kcal
Maltodextrin M 100	25.9	103.5	20%	0.0	0.0	0%	0.0	0.0	0%
Sugar, confectioners/powdered	18.0	70.2	14%	26.9	104.7	20%	26.9	104.7	20%
Milk, nonfat/skim, dry	11.0	42.1	8%	11.0	42.1	8%	11.0	42.1	8%
Protein, whey, concentrate	6.0	24.0	5%	6.0	24.0	5%	6.0	24.0	5%
Dried Cream Powder	5.5	39.6	8%	5.5	39.6	8%	5.5	39.6	8%
Pea Protein	2.2	8.0	2%	2.2	8.0	2%	2.2	8.0	2%
Oil, soybean, refined	24.0	212.2	41%	0.0	0.0	0%	0.0	0.0	0%
Oil, Shea nut	0.0	0.0	0%	5.0	44.2	8%	5.0	44.2	8%
Oil, canola	0.0	0.0	0%	25.0	221.0	42%	20.0	176.8	34%
Oil, flaxseed	0.0	0.0	0%	0.0	0.0	0%	5.0	46.4	9%
Oil, soybean lecithin	2.5	19.1	4%	2.5	19.1	4%	2.5	19.1	4%
Cocoa, powder, unsweetened	0.0	0.0	0%	10.0	22.8	4%	10.0	22.8	4%
Vitamin mineral mix	3.8	0.0	0%	3.8	0.0	0%	3.8	0.0	0%
Total	100.0	518.6	100%	100.0	525.5	100%	100.0	527.7	100%
Lipid	30.8	270.3	52%	38.2	335.7	64%	38.2	336.5	64%
Carbohydrate	50.8	203.4	39%	40.6	162.4	31%	40.6	162.4	31%
Protein	11.5	45.8	9%	13.4	53.6	10%	13.4	53.6	10%

SB: Shea butter; SB + F: Shea butter with flax seed oil.

**Table 2:** Fatty acid composition in LNS products (g/100 g).

Fatty Acid ^1^	A-20	SB	SB + F
16:0	2.7	1.2	1.3
16:1	0.0	0.1	0.1
9c-16:1	0.0	0.4	0.3
18:0	1.1	2.5	2.9
18:1 Total	5.5	13.3	12.1
Undefined 18:2	12.9	4.7	4.6
Undefined 18:3	2.0	5.0	6.6
20:0	0.1	0.4	0.4
20:1 Total	0.0	2.3	1.8
Total Saturated fat	3.9	4.1	4.6
Total Monounsaturated fat	5.6	16.0	14.2
Total Polyunsaturated fat	14.9	9.8	11.2
ω-3/ω-6 ratio	0.2	1.1	1.4

^1^ Calculations based on Firestone [39].
